# Quantifying Demyelination in NK venom treated nerve using its electric circuit model

**DOI:** 10.1038/srep22385

**Published:** 2016-03-02

**Authors:** H. K. Das, D. Das, R. Doley, P. P. Sahu

**Affiliations:** 1 Department of Electronics and Communication Engg., Tezpur University, Tezpur-784028, Assam, India; 2 Department of Molecular Biology and Biotechnology, Tezpur University, Tezpur-784028, Assam, India

## Abstract

Reduction of myelin in peripheral nerve causes critical demyelinating diseases such as chronic inflammatory demyelinating polyneuropathy, Guillain-Barre syndrome, etc. Clinical monitoring of these diseases requires rapid and non-invasive quantification of demyelination. Here we have developed formulation of nerve conduction velocity (NCV) in terms of demyelination considering electric circuit model of a nerve having bundle of axons for its quantification from NCV measurements. This approach has been validated and demonstrated with toad nerve model treated with crude *Naja kaouthia* (NK) venom and also shows the effect of Phospholipase A_2_ and three finger neurotoxin from NK-venom on peripheral nerve. This opens future scope for non-invasive clinical measurement of demyelination.

Speedy and efficient transmission of action potential sequences carrying neuro-signals depends on axon membrane sheath made by myelin^1^,^2^. Demyelination in nerve axon of peripheral nerve results in reduction of nerve conduction velocity (NCV) occurred in most of neuro diseases such as chronic inflammatory demyelinating polyneuropathy (CIDP)^3^,^4^, Guillain-Barre syndrome (GBS)^5^,^6^ etc. In fact, Node of Ranvier (NoR) is distorted by demyelination leading to slow movement of Na^+^/K^+^ ions. Clinical analysis of these diseases requires quantification of demyelination. In this direction, invasive measurement of demyelin/myelin thickness using SEM imaging and nerve biopsy is a destructive and clinically critical and harmful for the patients. Moreover, these techniques have risk for site selection of the damaged nerve and may cause discomfort and infection after surgery for nerve testing^7^,^8^. Hodgkin and Huxley first demonstrated quantitative description of axonal membrane currents for giant axon of a squid with the use of electrical circuit model developed by them^9^. Later, experiments were performed on toad model with consideration of node of Ranvier to prove that the membrane currents are dependent on Na^+^, K^+^ ions and other Ca^++^ ions (contributing leakage current). As seen in previous studies^10^,^11^,^12^, snake venom especially *Naja kaouthia* venom causes degradation of myelin sheath due to having high percentage of phospholipase A_2_ (PLA_2_), neurotoxins and cytotoxins^13^,^14^,^15^,^16^. In fact, NK-PLA_2_ belonging to lypolytic enzyme family catalyzes the hydrolysis of fatty acid esters at position 2 of 1, 2 diacyl-sn-phosphoglycerides producing lysophospholipids and free fatty acids^17^. So *Naja kaouthia* venom is one of the strong candidates for demyelination of myelin sheath due to the presence of NK-PLA_2_. Previous studies show that PLA_2_ also causes Alzheimer’s disease^18^, Multiple Scelerosis (MS)^19^,^20^, Epilepsy^21^ due to motor dysfunction. In this direction the development of toad nerve model has revolutionized the field of neuroscience especially for human peripheral clinical treatment^22^,^23^. Moreover, the membrane transport and activity of neurotransmitter are better examined on isolated toad nerve having bundle of axons^24^,^25^.

## Introduction

### Electric model of a demyelinated nerve

**Figure 1:**
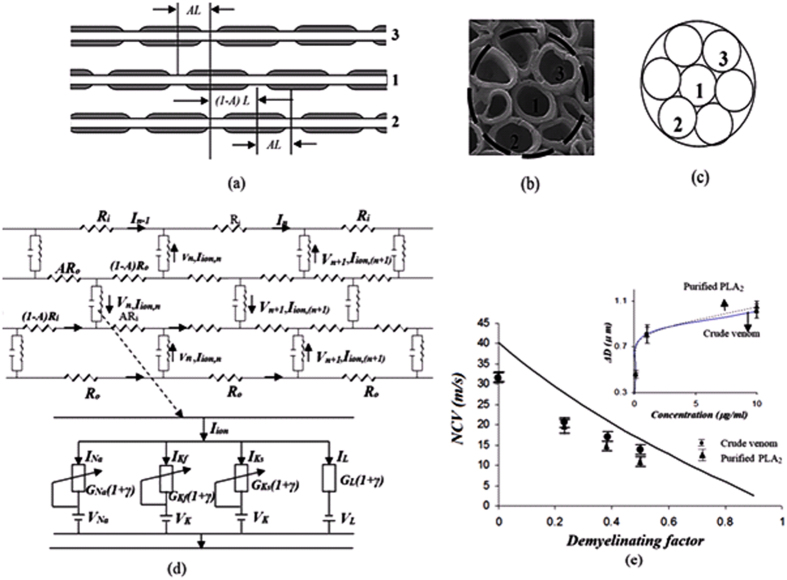
A demyelinated nerve consists of bundle of axons. (**a**) a bundle of nerve denoting three nerves – nerve 1, nerve 2 and nerve 3 respectively where the Node of Ranvier of axons are misaligned by alignment factor A (where 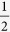 ≤ A ≤ 1). A = 1 indicates that two axons are aligned exactly, and 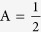 indicates that two axons are evenly staggered. “L” is the internodal length i.e., the distance between two internodes in the nerve. (**b**) Typical SEM image of normal nerve having bundle of axons in which axon 1 is surrounded by six axons. (**c**) Correspondingly to SEM image we consider axon 1 is surrounded by six axons of equal diameter. We consider only ephaptic interactions of six surrounded axons on neuro conduction in axon 1. (**d**) Electric circuit model corresponds to bundle of axons in a nerve shown in the figure. (**e**) Nerve conduction velocity (NCV in m/s) versus demyelinating factor is obtained by using equation (1). NCV is formulated by using electric circuit model of a nerve having bundle of axons misaligned by alignment factor, 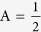. The NCV of toad sciatic nerve decreases with increase of demyelination. The experimental points of black dots are obtained from conduction of action potential (latency between proximal and distal action potential) in sciatic nerve of frog model demyelinated by *Naja kaouthia* crude venom with concentration 0.1, 1.0 and 10 μg/ml. Similarly experimental points of triangle are obtained from sciatic nerves treated with NK-PLA_2_ (purified from crude venom). The inset of Fig. 1(e) shows demyelination ΔD in μm versus concentration of crude venom/NK-PLA_2_ (ΔD is the difference between normal nerve thickness and demyelinated nerve, obtained from SEM images shown in Fig. 2). The solid line and dashed line in inset figure are drawn by using experimental points with minimum deviation. The lines are almost close to each other proving NK-PLA_2_ mainly responsible for demyelination of the nerve of toad model.

A rigorous theory of electrical circuit model of a demyelinated nerve was made by previous authors^26^,^27^,^28^. Since frog nerve consists of bundle of axons (Fig. 1(a,b)), H-H electrical circuit model for giant nerve is modified considering bundle of axons as shown in Fig. 1(b,c). The ephaptic interactions between action potential impulses of parallel axons in a bundle is demonstrated to describe NoR misalignment among axons (Fig. 1(a,d)). The nerve conduction velocity with incorporation of demyelinating factor γ, alignment parameter A and an ephaptic coupling constant α among axon-1 surrounded by six axons derived as


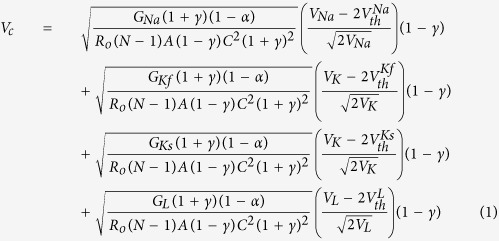


where, N = number of nerves surrounding a demyelinated nerve (here N is considered to be six), as shown in Fig. 1(b,c),
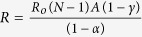
, 
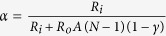
,

*γ* = demyelination factor which is defined as the ratio of change in myelin thickness due to demyelination to the actual amount of myelin thickness of the nerve. The effect of demyelination factor upon resistance, capacitance and conductance in the circuit has already been discussed in our previous work^29^,^30^. The total resistance in the circuit is mainly divided into internal resistance (R_i_) and external resistance (R_o_). The I_n_ and V_n_ are the ionic current and voltage at node n. The total ionic current I_ion,n_ at node n consists of current contributed by sodium ion as (I_Na,n_), fast potassium as (I_Kf,n_), slow potassium as (I_Ks_,_n_) and also leakage current contributed by mainly calcium and chlorine as (I_L,n_), through demyelinated ionic resistances 1/G_Na_(1 + γ), 1/G_Kf_ (1 + γ), 1/G_Ks_(1 + γ) and 1/G_L_(1 + γ) respectively, where G_Na_, G_Kf_, G_Ks_ and G_L_ are ionic conductance of sodium ion, fast potassium, slow potassium and leakage ions (mainly calcium, chlorine etc.). The voltage V_n_ at node n depends on V_Na_, V_Kf_, V_Ks_ and V_L_, Nernst (diffusion) potentials of sodium, fast potassium, slow potassium and leakage ions at which corresponding currents return to be zero. V_th_^Na^, V_th_,^Kf^, V_th_^Ks^ and V_th_^L^ are threshold voltages of sodium, fast potassium, slow potassium and leakage currents respectively at which sodium current, fast potassium, slow potassium and leakage currents start to flow at active node as shown in Fig. 1(d). The values of ionic conductance, Nernst potential and threshold voltage of sodium, slow potassium, fast potassium and leakage ions of frog are obtained from previous works^29^,^30^. The variation of NCV is determined by using equation (1), decreases with increase of demyelinating factor (Fig. 1(e)). To validate our formulation of neuro-signal conduction, the experiments were performed on toad nerves treated with crude venom of *Naja kaouthia* (NK) for six times and the decrease of myelin sheath (demyelination) with increase of concentration of crude venom was observed every time (Fig. 1(e)).

## Results

**Figure 2:**
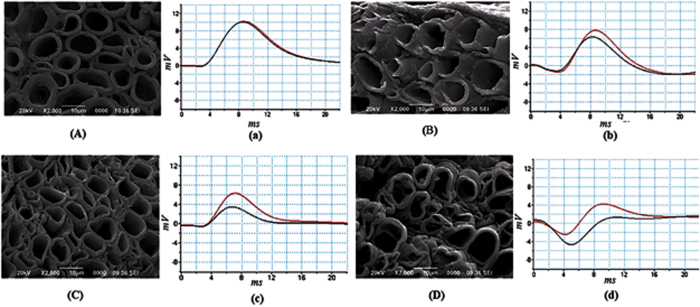
Effect of crude venom with different concentration on sciatic nerves of toad and their nerve conduction signals (proximal and distal action potential) with corresponding SEM images of their sciatic nerves. We have repeated the experiments for six times and estimated results statistically (as shown in supplementary table S1 and supplementary Fig. S1(a)). (**A**) SEM image of normal sciatic nerves with myelin thickness of 1.79 ± 0.23 μm at *γ* = 0 (normal). (a) Corresponding neuro conduction signal obtained by AD instrument (Powelabs, Australia) consists of proximal and distal action potential. The NCV is determined by using distance between two electrodes and divided by latency between proximal and distal action potential (shift between peaks of proximal and distal action potential) as 32.1 ± 1.71 m/s, which is very close to normal value of toad nerve. (**B**) SEM image of toad sciatic nerve treated with 0.1 μg/ml crude NK-venom shows slight reduction of myelin sheath, the thickness measured is 1.22 ± 0.15 μm corresponding to demyelinating factor, *γ* = 0.32 (which represents 32% reduction of myelin thickness). (b) Corresponding neuro conduction signal provides NCV of 21.95 ± 0.99 m/s (determined from latency between proximal and distal action potential peaks) which is slight less than the normal value. (**C**) SEM image of toad sciatic nerve treated with 1 μg/ml crude NK-venom shows more reduction of myelin thickness of 1.00 ± 0.9 μm (i.e., more demyelination) with *γ* = 0.44 (which indicates 44% reduction of myelin thickness). (c) NCV is determined from neuro conduction signal as 18.33 ± 1.2 m/s which is less than normal value. (**D**) SEM image of toad sciatic nerve treated with 10 μg/ml crude NK-venom shows reduction of myelin sheath, the myelin thickness of which is found to be 0.91 ± 0.08 μm corresponding to demyelinating factor, *γ* = 0.49 (which shows 49% reduction of myelin thickness). (d) NCV is estimated from latency of neuro conduction signal as 14.28 ± 0.85 m/s which is far below normal value.

**Figure 3:**
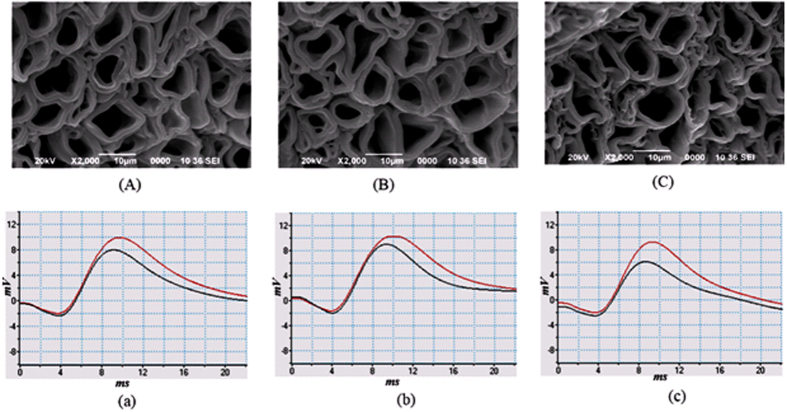
Effect of purified NK-PLA_2_ with different concentration on sciatic nerves of toad and their nerve conduction signals (proximal and distal action potential) with corresponding SEM images of their sciatic nerves. We have performed the experiments repeatedly and the myelin thickness with its demyelinating factor is estimated statistically (as shown in supplementary table S1 and supplementary Fig. S1(b)). (**A**) SEM image of sciatic nerve with 0.1 μg/ml NK-PLA_2_ with myelin thickness of 1.4 ± 0.29 μm with *γ* = 0.21 (which shows 21% reduction of myelin thickness). (a) Corresponding neuro conduction signal obtained by AD instrument and NCV is obtained from distance between two electrodes divided by latency between proximal and distal action potential (shifts between peaks of proximal and distal action potential). (**B**) SEM image of toad sciatic nerve treated with 1 μg/ml NK-PLA_2_ shows a reduced thickness of myelin of 1.12 ± 0.34 μm corresponding to demyelinating factor, *γ* = 0.37 (which represents 37% reduction of myelin thickness). (b) Corresponding neuro conduction signal. (**C**) SEM image of toad sciatic nerve treated with NK-PLA_2_ concentration of 10 μg/ml with more demyelination, the myelin thickness being 0.89 ± 0.15 μm, the demyelinating factor being *γ* = 0.5(which indicates 50% reduction of myelin thickness). (c) Corresponding neuro conduction signal at 10 μg/ml of NK-PLA_2_. The SEM images shows increase of demyelination with increase in purified NK-PLA_2_ concentration, while the neuro conduction signals confirms the reduction of NCV as the concentration of purified NK-PLA_2_ increases.

For experimental estimation of demyelination, sciatic nerves were isolated from toad and its storage was made in Ringer’s solution. During experiments on nerve conduction by using AD instruments, the sciatic nerves were kept moist in nerve chamber using Ringer’s solution. The reduction of myelin thickness (i.e., demyelination) with increase of crude venom concentration was confirmed by SEM images in Fig. 2(A–D). We have obtained experimentally propagation of action potential stimuli applied on sciatic nerves treated with different crude venom concentration as shown in Fig. 2(a–d). The nerve conduction signals obtained from proximal and distal action potential nerve treated with 0.1 μg/ml, 1.0 μg/ml and 10 μg/ml crude venom concentration shows reduction of nerve conduction velocity (NCV) due to demyelination of axons in the nerve (Fig. 2). It also shows the reduction of action potential amplitude with crude venom concentration. The experimental values of NCV in Fig. 1(e) matches well with theoretical values of NCV which was obtained by using the formulation (equation 1) using MATLAB and these values of NCV may used to predict the amount of demyelination as shown in the curve of the figure. From the SEM images (Fig. 2(B–D)), the demyelination factor *γ* of sciatic nerve treated with 0.1 μg/ml, 1.0 μg/ml and 10 μg/ml crude venom concentration are estimated as ~0.21, 0.37 and 0.5 respectively (as shown in supplementary table S1 and supplementary Fig. S1(a)) which are close to those obtained from NCV results of Fig. 1(e). Estimation of demyelination from NCV formulation will avoid the invasive difficulties of SEM imaging and nerve biopsy. The rate of increase of demyelination (ΔD) with crude venom concentration is very fast up to 1 μg/ml and it becomes saturated after that (inset of Fig. 1(e)). As seen in Fig. 3, NK-PLA_2_ present in crude venom is mainly responsible for demyelination as per the following reaction of NK-PLA_2_ with phospholipids of myelin sheath.


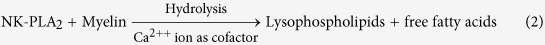


The rate of reaction increases with increase of NK-PLA_2_ molecules (as NK-PLA_2_ concentration increases with crude venom concentration/dose) and as a result more demyelination takes place with increase in concentration of NK-PLA_2_ (Fig. 3). The nerve conduction experiment of NK-PLA_2_ (purified from NK crude venom) treated sciatic nerves shows reduction of NCV due to demyelination in which the variation of demyelination thickness (ΔD) with NK-PLA_2_ concentration for NK-PLA_2_ treated nerve is almost close to that of crude venom treated nerve proving major contribution of demyelination by NK-PLA_2_ (Fig. 1(e)). The demyelination factor *γ* of sciatic nerve (treated with 0.1 μg/ml, 1.0 μg/ml and 10 μg/ml NK-PLA_2_ concentration) estimated from the SEM images (Fig. 3(A–C), are also almost close to those obtained from NCV results of Fig. 1(e) as shown in supplementary table S1 and supplementary Fig. S1(b).

The similar results are obtained after repeated nerve conduction experiments of normal sciatic nerve and nerves demyelinated with both crude venom and NK-PLA_2_ and NCV are estimated with ±SD (standard deviation) statistically to minimize the percentage of error as per standard method^31^. The theoretical curve of NCV versus demyelination factor (Fig. 1(e)) validated with nerve conduction experiments, establish a hypothesis to quantify demyelination in the patients and the demyelination of nerve is obtained from known NCV using demyelination factor as shown in the curve. This hypothesis may help the medical experts to estimate the quantity of demyelination from the range of NCV of the patients.

### Reduction of amplitude of NC signal

**Figure 4:**
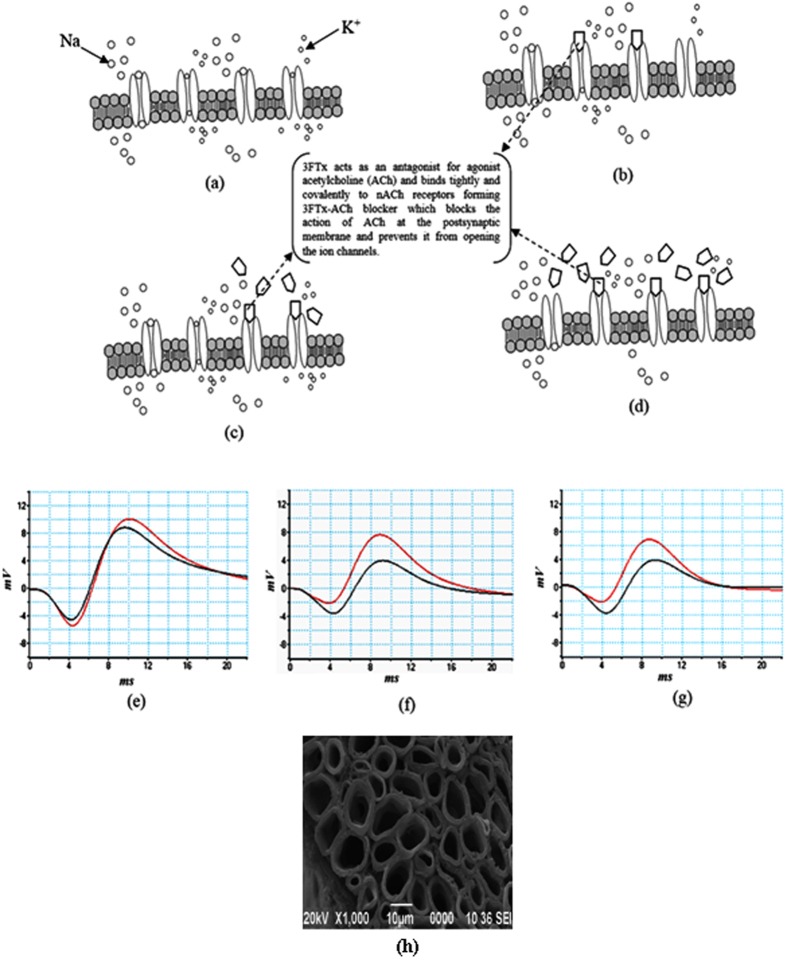
Mechanism of channel blocking in cell membrane. We have performed channel blocking of sciatic nerve using 3FTx neurotoxin for 5–6 times and the results obtained from repeated experiments are almost same. We have shown the results which are estimated statistically. (**a**) transportation of Na^+^ into cell through cell membrane and K^+^ transportation leaving from cell through membrane and provides saltatory movement of the nerve impulse (action potential). (**b**) Shows less blocking of Na^+^ and K^+^ channel by NK crude venom in which 3FT weak neurotoxin binds with acetylcholine receptors (AchR) and then blocks channel. Number of blocking is small if crude venom concentration is less. (**c**) Represents more blocking if crude venom concentration becomes more because of presence of more 3FT neurotoxin. (**d**) Represents most of channels are blocked if crude venom concentration is very high (more than 10 μg/ml). (**e**) Neuro conduction signal (proximal and distal action potential) obtained by using AD instruments. There is a reduction of peak of distal action potential with respect to proximal action potential showing Na^+^ and K^+^ channel blocking. (**f**) Neuro conduction signal of 1 μg/ml 3FTx-weak neurotoxin treated sciatic nerve shows a sharp reduction of peak of action potential. (**g**) Neuro conduction signal of 10 μg/ml 3FTx neurotoxin shows almost same sharp reduction of distal action potential peak. As in 1 μg/ml 3FTx treated sciatic nerve, most of channels are blocked (Fig. 4(d)), same reduction of distal action potential was observed even after treatment of 10% 3FTx treated sciatic nerve. (**h**) SEM image of toad sciatic nerve treated with 10 μg/ml 3FTx shows no reduction of myelin sheath even at high concentration. The myelin thickness of which is found to be 1.74 ± 0.18 μm, which is similar to normal sciatic nerve.

The Nerve conduction signals in Fig. 4(e–g) shows decrease of amplitude of wave due to both Na^+^ and K^+^ channel blocking in crude venom treated toad nerve. The weak three finger neurotoxin (3FT-neurotoxin) which constitutes a major percentage of cobra venom is mainly responsible for channel blockade activity in nerve axon^15^,^32^,^33^,^34^,^35^,^36^. 3FT-neurotoxin binds the neural Ach receptors causing blocking of transmission of both Na^+^ and K^+^ ions through the ion gated channels (Fig. 4(a–d)). The blockade increases with increase of crude venom concentration/dose due to increase in 3FTx concentration. The nerve conduction experiments of 3FT-neurotoxin (purified from crude venom) treated nerve confirms reduction of action potential due to blocking of channel^37^. There is no change of value of latency between peak of proximal and peak of distal action potential even with increase of 3FTx concentration proving no reduction of myelin thickness of sciatic nerve treated with 3FTx and it is confirmed from SEM image (Fig. 4(h)).

## Discussion

Although we have performed experiments on sciatic nerve isolated from toad our findings *may* open a non-invasive clinical way to quantify demyelination of a peripheral nerve from NCV measurements. The observation shown here is emerging experimental evidences for demyelination and channel blockade using NK crude venom. Our electric circuit model for a nerve consisting of six axons matches with experimental results of demyelination by using NK crude venom. Our results may also provide demyelination analysis of different critical neuro-diseases such as CIDP, GBS etc. Although our experiments are limited to peripheral sciatic nerve, our model may also quantify demyelination of central nervous system (CNS) including motor nerve from nerve conduction velocity. So, future studies will be needed to determine the role of crude venom in CNS demyelination. The result may extend to support non-invasive clinical analysis of CNS demyelinating diseases like multiple sclerosis, Alzheimer’s disease, Epilepsy, etc. In our works, we have also demonstrated channel blocking in sciatic nerve using 3FTx weak neurotoxin (Fig. 4). It may lead a way to quantify Na^+^ and K^+^ channel blocking, due to the binding of 3FTx with nACh receptors.

## Materials and Methods

### Ethics Statement

Common Asian toads (*Duttaphrynus melanostictus*) were used for neurotoxicity studies. All animal experiments were performed in accordance with the guidelines from the Defence Research Laboratory, Tezpur, Assam (India) under Registration Number 1227/bc/07/CPCSEA and approved by Tezpur University Animal Ethical Committee (TUAEC) (Approval no: DORD-Pro/TUAEC/10-56/14/Res-06). Efforts were made to minimize the number as well as sufferings of animals during the experiments.

### Animals

For validation of NCV in a demyelinated nerve, we have extracted sciatic nerve from common Asian toad (*D. melanostictus*). Further, we have attempted to see the variation of demyelination in the sciatic nerve using different concentration of snake venom. We have taken 48 toads of same age (~1–2 months) for validation of demyelination measurements from nerve conduction velocity and divided into four groups (A, B, C and D). The sciatic nerves of toads of group A is not treated with crude venom whereas the sciatic nerves of B,C and D groups are treated with *Naja kaouthia* venoms of 0.1 μg/ml, 1 μg/ml and 10 μg/ml respectively.

### Collection of snake venom

Crude venom from *Naja kaouthia* of North East India was collected from the wild and stored as explained in Das *et al*.^38^. The permission for milking of snakes from Assam was obtained from Principal Chief Conservator of Forest (Wild Life) and Chief Wild Life Warden of Assam, India (WL/FG.27/tissue Collection/09 dated 07/10/2011).

### Determination of protein content

Protein concentration of the crude venom and purified toxin was determined according to Lowry’s method using BSA as a standard^39^.

### Partial purification of fraction containing Phospholipase A_2_ (PLA_2_) and three finger toxin (3FTx)

PLA_2_ and three finger toxin was purified as described in Das *et al*.^37^. Briefly, crude venom was subjected to single step fractionation on RP-HPLC using symmetry C18 column (5 μ, 4.6 × 250 mm, 300 Å) (Waters, USA). Fractionation was carried out in a linear gradient of buffer B (80% ACN containing 0.1%TFA) in a pre-equilibrated column with buffer A (0.1% TFA in milli Q water) on a HPLC system (Waters, USA). Eluted protein peaks were monitored at 215 and 280 nm and collected manually. Considering the retention time of the protein peaks likely to contain PLA_2_ and 3FTxs were isolated.

### Phospholipase A_2_ activity assay

PLA_2_ activities of the crude venom as well as the fractionated peaks were determined according to Doley and Mukherjee^40^ using egg yolk as a substrate. One unit of PLA_2_ activity was defined as the amount of protein which produces a decrease in 0.01 absorbance in 10 mins at 740 nm.

### Preparation of Sciatic nerve

The method as described by Katsuki *et al*. was followed for sciatic nerve preparation^31^. In brief, common Asian toads *(Duttaphrynus melanostictus)* weighing 30–35 gm of either sex were decapitated and then pithed. Sciatic nerve of length 4–6 cm and 0.5–1 mm diameter was dissected from lumber plexus to the knee joint. Throughout the procedure the nerve was continuously flooded with Ringer’s solution. Finally, the dissected nerve was treated with venom sample and mounted on nerve chamber (AD Instruments, Powerlabs, Australia) containing Ringer’s solution.

### Recording of compound action potential (CAP) and determination of nerve conduction velocity (NCV)

**Figure 5:**
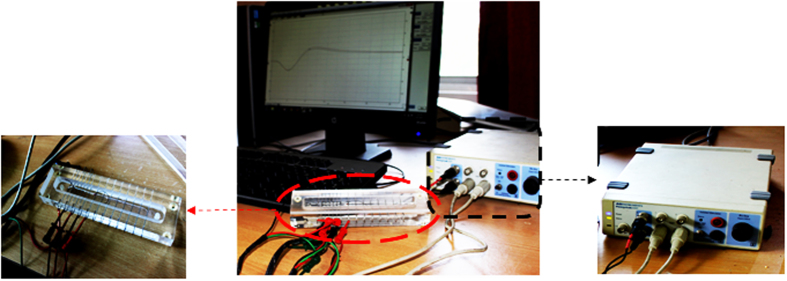
A schematic picture of the recording set up. AD Instrument consists of a dual Bio Amp/stimulator and a nerve chamber. The dual Bio Amp/stimulator is used to obtain and record CAP and NCV. The nerve chamber is equipped with 15 stainless steel electrodes and the dissected nerve is mounted on it filled with Ringer’s solution. The setup was comprised of two male BNC (Bayonet Neill–Concelman) connectors to three micro-hooks constructed of gold-plated beryllium copper which was used to stimulate the nerve. CAP was analyzed by SCOPE (Powerlabs, Australia).

Various concentrations of crude NK venom and partially purified PLA_2_ from the crude venom (0.1 μg/μl, 1 μg/μl and 10 μg/μl) was treated *in-vitro* to the dissected nerve for 2 mins. Standard techniques for extracellular recordings were followed. CAP was measured in a nerve chamber (AD Instruments, Powerlabs, Australia) equipped with 15 stainless steel electrodes as shown in Fig. 5. A dual Bio Amp/stimulator was used to obtain and record CAP. In brief, the dissected sciatic nerve was externally stimulated with a frequency of 1 Hz where pulses at 0.1 ms duration were used to determine the CAP^31^. The setup was comprised of two male BNC (Bayonet Neill–Concelman) connectors to three micro-hooks constructed of gold-plated beryllium copper which was used to stimulate the nerve. The electrodes for proximal and distal stimulus recording were placed at a distance of 3 cm. Nerve end with lumber plexus of spinal cord was connected with proximal recording electrode and electrode at nerve end connecting knee joint acted as distal recording electrode. Each experiment for recording of CAP was completed within 20 s of timeframe to avoid drying of the dissected nerve. CAP was analyzed by SCOPE (Powerlabs, Australia). Nerve only treated with Ringer’s solution was considered as control. The experiments were performed six times and ±SD was estimated statistically to minimize the percentage of error as per standard method^31^. The NCV was calculated using the latency and distance data from the signal.

### SEM imaging of sciatic nerve treated with different concentration of venom

Sciatic nerves were subjected for SEM analysis to check the effect of crude venom on morphology of the isolated nerve. Untreated nerve is served as control for the analysis. Sciatic nerve preparations were incubated for 15 mins with various concentrations of crude venom (0.1 mg/ml–10 mg/ml) and primary fixation was done using 2.5% gluteraldehyde for 4 hr. Further the nerve was subjected for secondary fixation using 1% OsO_4_ (Osmium tetroxide) for 4 hr for better penetration. Cross section of sciatic nerve was made by slicing at a length of 10 mm using glass cutter in a microtome maintaining uniformity. The sliced nerve segments were further observed under SEM for the structural change. Demyelination or reduction of myelin thickness considering 6–7 nerves in a bundle was quantified with the use of measuring scale installed in the SEM.

## Additional Information

**How to cite this article**: Das, H. K. *et al*. Quantifying Demyelination in NK venom treated nerve using its electric circuit model. *Sci. Rep*. **6**, 22385; doi: 10.1038/srep22385 (2016).

## Supplementary Material

Supplementary Information
