# Supplementary material for: Quantifying Demyelination in NK venom treated nerve using its electric circuit model
Source: Sci Rep. 2016 Mar 2;6:22385. doi: 10.1038/srep22385 (PMC4773768; doi:10.1038/srep22385)
Supplement: Supplementary Information [file srep22385-s1.doc]

**Quantifying Demyelination in NK venom treated nerve using its electric circuit model**

H. K. Das^1^, D. Das^2^, R. Doley^2^ and P. P. Sahu^1*^

**Supplementary table S1.** Demyelinating factor (γ) of a normal nerve and nerves treated with three different concentrations of crude venom and Nk-PLA_2_. γ was estimated by calculating the ratio of change in myelin thickness due to demyelination to the actual amount of myelin thickness of the nerve and the experiment was performed for six times with normal nerves and nerves treated with 0.1µg/ml, 1.0µg/ml and 10µg/ml of crude venom and Nk-PLA_2_ respectively.

| **Sample** | **Demyelinating factor (γ)** | | | | | | |
| --- | --- | --- | --- | --- | --- | --- | --- |
|  | **Experiment** | **I** | **II** | **III** | **IV** | **V** | **VI** |
| **Normal Nerve** | | 0 | 0 | 0 | 0 | 0 | 0 |
| **Crude venom** | 0.1μg/ml | 0.3271 | 0.3220 | 0.3184 | 0.3111 | 0.3186 | 0.3128 |
|  | 1.0μg/ml | 0.4406 | 0.4406 | 0.4469 | 0.4333 | 0.4341 | 0.4413 |
|  | 10μg/ml | 0.4972 | 0.4972 | 0.4972 | 0.4889 | 0.4890 | 0.4810 |
| **Nk-PLA_2_** | 0.1μg/ml | 0.2203 | 0.2147 | 0.2179 | 0.2167 | 0.2198 | 0.2179 |
|  | 1.0μg/ml | 0.3785 | 0.3785 | 0.3743 | 0.3667 | 0.3681 | 0.3743 |
|  | 10μg/ml | 0.5141 | 0.5141 | 0.5028 | 0.4944 | 0.4945 | 0.4972 |


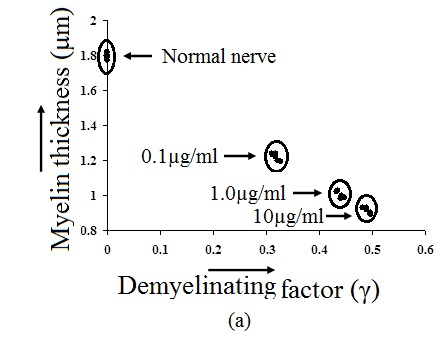

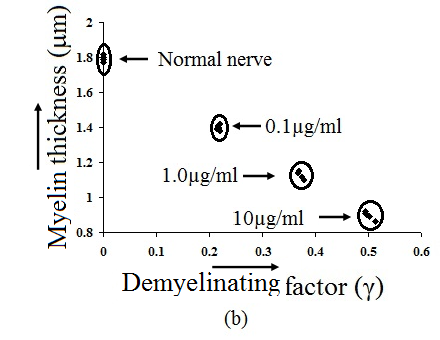


**Supplementary figure S1.** Demyelinating factor (γ) versus myelin thickness (µm). (a) γ was estimated for normal nerves and nerves treated with 0.1µg/ml, 1.0µg/ml and 10µg/ml of crude venom. The black dots represent the demyelinating factor obtained from six different experiments performed on normal nerves and nerves demyelinated with three different concentrations of crude venom. (b) Estimated γ for normal nerves and nerves treated with 0.1µg/ml, 1.0µg/ml and 10µg/ml of Nk-PLA_2_. As seen in (a), the black dots represent the demyelinating factor obtained from six different experiments performed on normal nerves and nerves demyelinated with three different concentrations of Nk-PLA_2_.
